# Liver and brain differential expression of one-carbon metabolism genes during ontogenesis

**DOI:** 10.1038/s41598-021-00311-9

**Published:** 2021-10-26

**Authors:** Apolline Imbard, Leslie Schwendimann, Sophie Lebon, Pierre Gressens, Henk J. Blom, Jean-François Benoist

**Affiliations:** 1 Biochemistry Hormonology Laboratory, Robert-Debré University Hospital, APHP, 48 bd Serurier, 75019 Paris, France; 2 LIPSYS2, Faculty of Pharmacy, Paris Saclay University, Chatenay-Malabry, France; 3 Université de Paris, NeuroDiderot, Inserm, Paris, France; 4 Centre for the Developing Brain, Department of Division of Imaging Sciences and Biomedical Engineering, King’s College London, King’s Health Partners, St. Thomas’ Hospital, London, UK; 5 Metabolic Unit, Department of Clinical Genetics, Center for Lysosomal and Metabolic Diseases, Erasmus MC, Rotterdam, The Netherlands

**Keywords:** Biochemistry, Molecular biology

## Abstract

One-carbon metabolism (1C metabolism) is of paramount importance for cell metabolism and mammalian development. It is involved in the synthesis or modification of a wide variety of compounds such as proteins, lipids, purines, nucleic acids and neurotransmitters. We describe here the evolution of expression of genes related to 1C metabolism during liver and brain ontogeny in mouse. The level of expression of 30 genes involved in 1C metabolism was quantified by RT-qPCR in liver and brain tissues of OF1 mice at E9, E11, E13, E15, E17, P0, P3, P5, P10, P15 developmental stages and in adults. In the liver, hierarchical clustering of the gene expression patterns revealed five distinct clades of genes with a first bifurcating hierarchy distinguishing two main developmental stages before and after E15. In the brain most of the 1C metabolism genes are expressed but at a lower levels. The gene expression of enzymes involved in 1C metabolism show dramatic changes during development that are tissue specific. mRNA expression patterns of all major genes involved in 1C metabolism in liver and brain provide clues about the methylation demand and methylation pathways during embryonic development.

## Introduction

One carbon metabolism (1C metabolism) supports multiple essential physiological processes. It comprises a series of interlinking metabolic pathways including the methionine and folate cycles that are central to cellular function, like methylation, and provide 1C units for the synthesis of DNA, polyamines, amino acids, creatine, neurotransmitters and phospholipids.

1C metabolism is essential from the very early stages of development to epigenetic programing of long-term development through its role in nucleic acid and histone methylation. The mammalian genome undergoes two extensive waves of reprogramming of CpG methylation patterns during embryogenesis, following fertilization (E4–E6 in mice) and after germline cell specification (from E14 to birth in mice)^1^.

**Figure 1:**
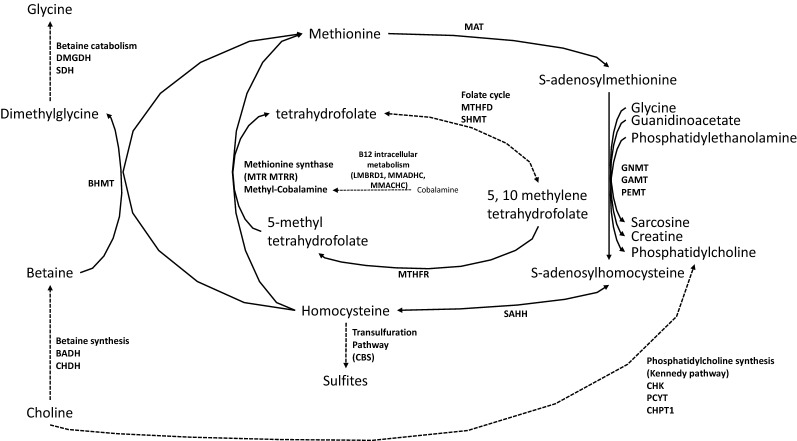
Schematic representation of 1C metabolism. *BADH* betaine aldehyde dehydrogenase, *BHMT* betaine-homocysteine methyltransferase, *CBS* cystathionine beta-synthase, *CHDH* choline dehydrogenase, *CHK* choline kinase, *CHPT1* choline phosphotransferase, *DMGDH* dimethylglycine dehydrogenase, *GAMT* guanidinoacetate methyltransferase, *GNMT* glycine N-methyltransferase, *MAT* methionine adenosyltransferase, *MTHFD* methylenetetrahydrofolate dehydrogenase, *MTR* methionine synthase, *MTRR* methionine synthase reductase, *PCYT* CTP-phosphocholine cytidyltransferase, *SAHH* S-adenosylhomocysteine hydrolase, *SDH* sarcosine dehydrogenase, *SHMT* serine hydroxymethyltransferase.

In mice, liver ontogeny initiates around embryonic day 9 (E9). During liver development, gene expression profiles change over time and determine the phenotypes and functions of liver^2–6^. Liver is a central organ for 1C metabolism, since about 85% of the methylation reactions take place in the liver. The importance of these reactions for the liver itself are illustrated by the liver diseases observed in most animal models of inactivation of 1C metabolism genes^7^. All the genes of the metabolic pathways that participate to the transfer of methyl groups such as the methionine and folates cycles but also the vitamin B12, betaine and choline metabolisms are highly expressed in liver (Fig. 1).

1C metabolism in the brain is among others required for creatine, serine and neurotransmitters synthesis but is also involved in the synthesis of phosphatidylcholine the major phospholipid in this tissue. The brain ontogeny begins early in mouse with the formation and closure of the anterior neuropore at E8 and the formation of the posterior neuropore at E9^8^. Next, the forebrain vesicle subdivides into telencephalic and diencephalic vesicles and Rathke's pouch and the nasal processes start to form.

1C metabolism is also involved in the pathophysiology of neural tube defects (NTD). In fact, a relation between folate and NTDs has been observed more than 30 years ago^9,10^. In the 90s, clinical randomized trials have proven that periconceptional folic acid supplementation can prevent occurrence^11^ and recurrence^12^ of NTDs. The underlying mechanisms by which folates contribute to reduction in NTD risks have not yet been clearly elucidated. One hypothesis is that folic acid prevents NTDs through their role in 1C metabolism^13^.

Despite the key role of 1C metabolism in liver and brain metabolism, little is known about the expression of the genes involved in this metabolism during development. There are only few and discordant data regarding the liver^14–16^ and only data for some genes at specific gestational ages for the brain^17^.

The purpose of this study is to provide, in mice, a comprehensive quantification of mRNA in the liver and brain tissues of a set of 30 genes involved in 1C metabolism during ontogenesis between 9 days of gestation and 15 days after birth.

## Material and methods

### Animal studies

The study was performed in accordance with local prescriptions and the *NIH Guide for the Care and Use of Laboratory Animals* and in compliance with the ARRIVE guidelines. The study received an approval from the Inserm Robert-Debré Bichat ethic Committee (N°2011-14/676-0053).

OF1 pregnant females were fed with standard rodent chow ad libitum and sacrificed by cervical dislocation at the stages of 9 days (E9), 11 days (E11), 13 days (E13), 15 days (E15), and 17 days (E17) of gestation. We randomly selected 3 embryos for each stage from multiple litters and dissected them to collect the liver and brain tissues that we immediately froze in dry ice. Tissues were also collected from 3 OF1 newborns, at the age of 0 days (P0), 3 days (P3), 5 days (P5), 10 days (P10) and 15 days (P15) following the same procedure.

### Quantitative PCR

We extracted total RNA from 5 to 10 mg of liver and brain tissues using the RNeasy Lipid Tissue Mini Protocol Kit from Qiagen according to the manufacturer instructions (Qiagen, Hilden, Germany). RNAs were quantified using a Nanodrop 2000 (Thermo scientific, Waltham, Maryland, USA). We performed RT-PCR using 1 µg of RNA with the iScript cDNA Synthesis Kit according to the manufacturer instructions (Biorad Laboratories, Hercules, California, USA). Newly primers were designed (supplemental data) for genes involved in the methionine metabolism (*MTHFR, MAT1A, MAT2A, MAT2B, MTR, MTRR, SAHH, CBS*), the phosphatidylcholine synthesis through the Kennedy pathway (*CHKA, CHKB, CHPT1, PCYT1A, PCYT1B, PCYT2*), the betaine metabolism (*BADH, CHDH, BHMT, BHMT2, DMGDH, SDH)*, the folate metabolism (*SHMT1, SHMT2, MTHFD1, MTHFD1L, MTHFD2*), the vitamin B12 metabolism (*MMACHC, MMADHC, LMBRD1*) and for the genes of the main methyltransferases (*GAMT, GNMT, PEMT*) (Supplementary Table S1). Quantitative-PCR was performed on a thermocycler CFX384 Biorad C1000 (annealing temperature 60 °C and 40 cycles) with SYBRgreen as fluorophore. A melting curve between 65 and 95 °C was performed to verify the quality of the amplification. Each sample was quantified in duplicate. *GAPDH* and *RPL13a* were used as reference gene in liver and brain, respectively. Results are expressed in ∆∆Ct.

### Statistical analysis

Differences between several groups were first sought by the Kruskal–Wallis test. If the test showed significant differences (p < 0.05), groups were tested in pairs by the Mann–Whitney test. Statistical analyses were performed using GraphPad Prism 5.01 (GraphPad Software, La Jolla, California, USA https://www.graphpad.com/scientific-software/prism/). Gene expression data from liver and brain ontogenesis were analyzed using a two-way hierarchical clustering method (GENE-E) using one minus Pearson correlation and displayed as a dendrogram.

## Results

### Expression of genes involved in the 1-C metabolism during liver ontogenesis

mRNA levels were detectable in pre and postnatal mouse livers for all tested gene excepted *MTHFD2L* and *PCYT1B*.

**Figure 2:**
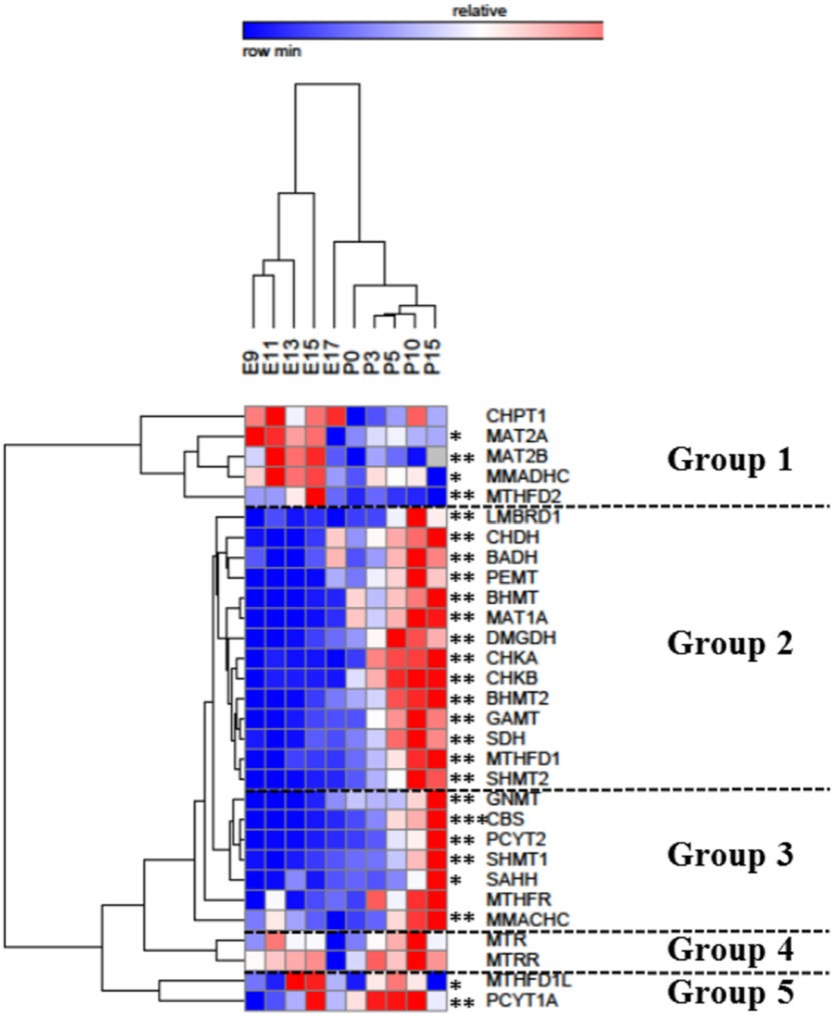
Two way hierarchical clustering of expression profiles for genes involved in 1C metabolism during liver ontogenesis. The two trees describe the relationship between different gene expression profiles (left tree) and various ages (upper tree). The dendrogram scale represents the correlation distances. Average expression values of three replicates per age are given by colored squares: red, relatively high expression; blue, relatively low expression. The dashed line categorizes the genes according to their expression profiles into five major groups. Stars represent the result of the Kruskall–Wallis test: *significant at the 0.05 level; **significant at the 0.01 level; ***significant at the 0.001 level.

Two ways hierarchical clustering of the gene expression patterns with respect to gestational age revealed 5 distinct clades of genes, respectively named group 1, 2, 3, 4 and 5 (Fig. 2).

Group 1 consisted of *CHPT1, MAT2A, MAT2B, MMADHC* and *MTHFD2*; group 2 included *LMBRD1, CHDH, BADH, PEMT, BHMT, MAT1A, DMGDH, CHKA, CHKB, BHMT2, GAMT, SDH, MTHFD1*, and *SHMT2*, group 3 included *GNMT, CBS, PCYT2, SHMT1, SAHH, MTHF* and *MMACHC*; group 4 consisted of *MTR* and *MTRR*, and group 5 of *MTHD1L* and *PCYT1A*.

**Figure 3:**
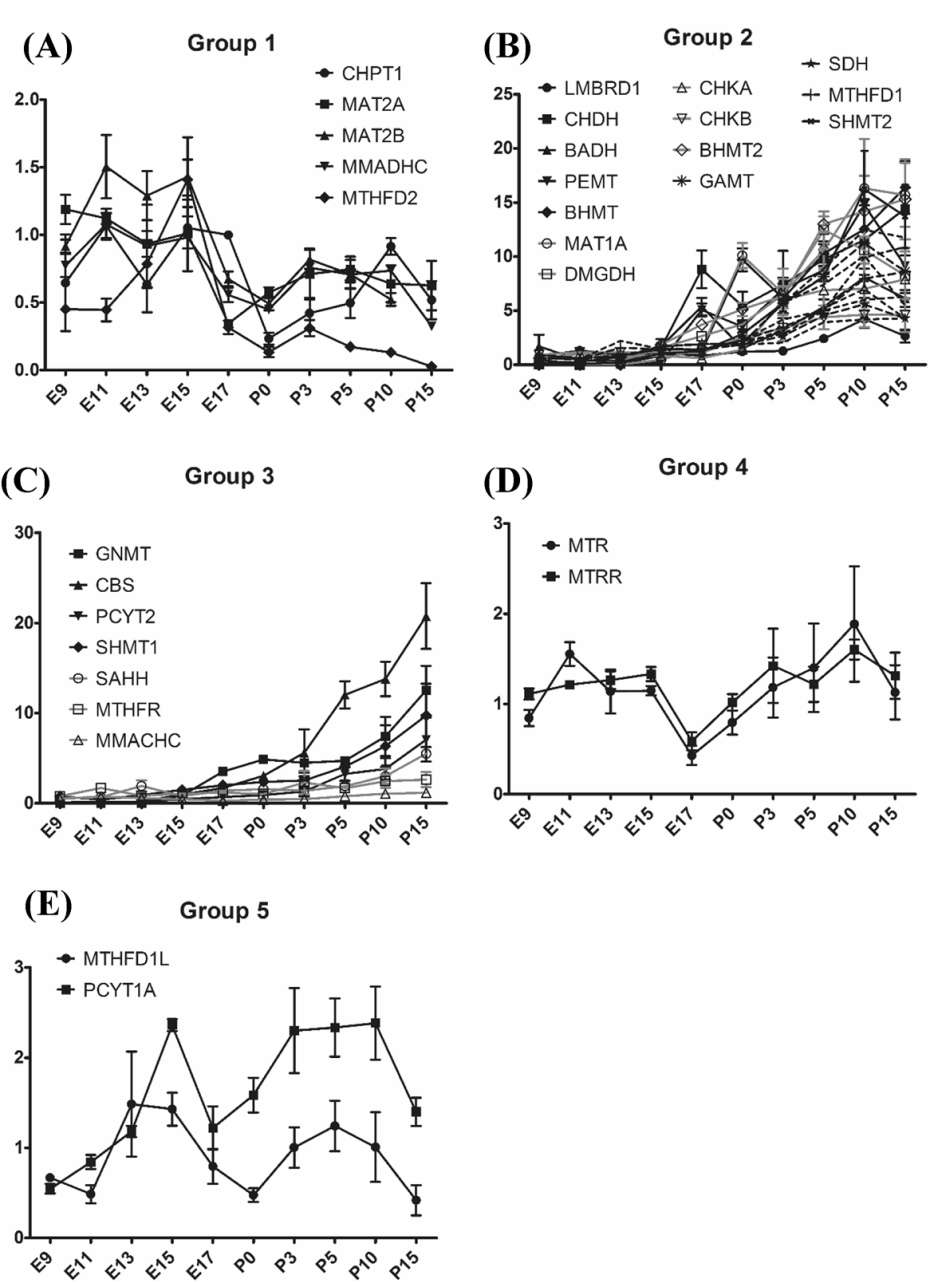
Variation of the expression of 1C metabolism genes in mouse liver ontogenesis. (**A**) Group 1 genes. (**B**) Group 2 genes. (**C**) Group 3 genes. (**D**) Group 4 genes. (**E**) Group 5 genes.

Group 1 gene expression is higher until E15 and decreased after to reach a minimum at birth (P0) and then have a moderate increased expression postnatally between P0 and P3 (Fig. 3A).

Group 2 genes have a really low level of expression before birth until E15, then increase exponentially since E17, reaching a plateau of maximal expression levels by 10 days of life (P10) (Fig. 3B). Among Group 2, *CBS* is the gene that revealed the highest increase of expression (around 2000 times between E9 and P15).

Group 3 genes have also a low expression during gestation with an exponential increase beginning at E17 up to the oldest age tested (P15) in contrast with group 2 (Fig. 3C).

Group 4 genes show a relatively stable low expression between E9 and E15, that decrease at E17 and then present a slight increase between E17 and P10 (Fig. 3D).

Group 5 genes present a gene expression that slightly increases between E11 and E15 then a decrease at E17 and re-increase slightly between E17 and P10 (Fig. 3E).

The clustering result on the time dimension shows that all time points remain in their original order (Fig. 2). According to these data, the largest correlation distance with respect to age is observed between embryonic day E15 and embryonic day E17. Samples between E9 and E15 are then subdivided into two sub-stages: E9–E13 and E15. Samples between E17 and P15 are also separated into 2 clades: E17 and P0–P15.

**Figure 4:**
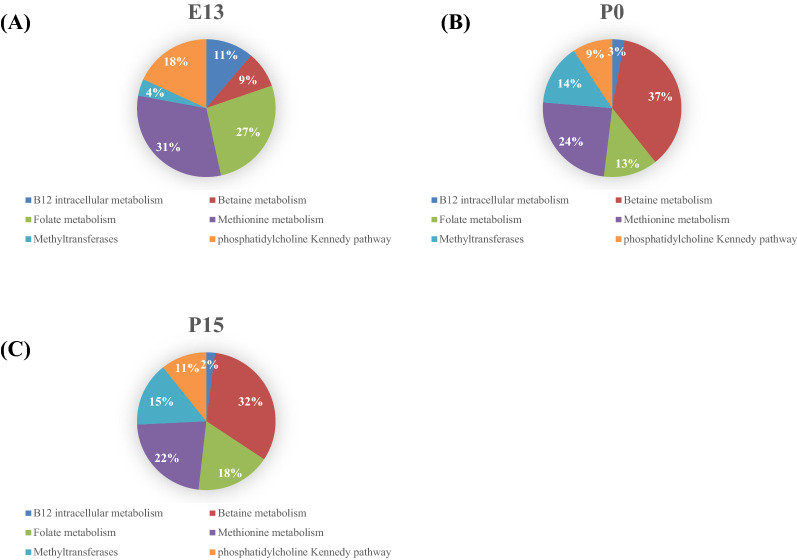
Percentages of liver mRNA expression of genes according to their metabolic pathway at a prenatal stage at embryonic day 13 (**A**), at birth (**B**) and at a postnatal stage, 15 days after birth (**C**).

Figure 4 shows the composition of 1C metabolism genes represented as percentage of the total 1C genes mRNA in a prenatal stage at embryonic day E13 (Fig. 4A), at birth (Fig. 4B) and in a postnatal stage at 15 days of life (Fig. 4C). At E13, the most abundant mRNA belong to the methionine metabolism and the folate metabolism. Between E13 and P0, the betaine metabolism became the predominant pathway for the gene expression. There were no significant differences in the relative abundance of gene expression between P0 and P15.

### Expression of genes involved in 1-C metabolism during brain ontogenesis

mRNA levels were detectable in pre and postnatal mouse brains for all tested gene excepted *MTHFD2L, PCYT1B and MAT1A*.

**Figure 5:**
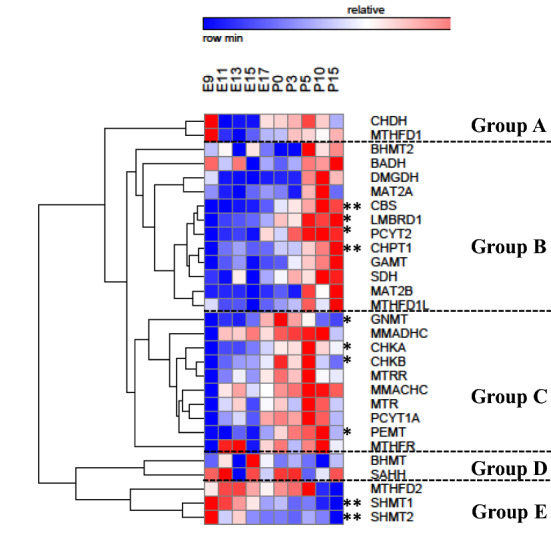
One-way hierarchical clustering of expression profiles for genes involved in 1C metabolism during brain ontogenesis. The left tree describes the relationship between different gene expression profiles. The dendrogram scale represents the correlation distances. Average expression values of three replicates per age are given by colored squares: red, relatively high expression; blue, relatively low expression. The dashed line categorizes the genes according to their expression profiles into five major groups. Stars represent the result of the Kruskall–Wallis test: *significant at the 0.05 level; **significant at the 0.01 level; ***significant at the 0.001 level.

Two ways hierarchical clustering of the gene expression patterns with respect to gestational age showed on the time dimension that E11 and E13 were exchanged and the largest correlation distance with respect to age is observed between embryonic day E15 and embryonic day E17 (data not shown). On the gene expression dimension, it revealed 5 distinct clades of genes, which we have named group A, B, C, D and E (data not shown). The one way hierarchical clustering of the gene expression patterns without the time dimension cluster analysis revealed the same 5 distinct clades of genes that the 2 ways hierarchical clustering (Fig. 5).

Group A consisted of *CHDH and MTHFD1*; group B included *BHMT2, BADH, DMGDH, MAT2A, CBS, LMBRD1, PCYT2, CHPT1, GAMT, SDH, MAT2B* and *MTHFD1L*, group C included *GNMT, MMADHC, CHKA, CHKB, MTRR, MMACHC, MTR, PCYT1A, PEMT* and *MTHFR*; group D consisted of *BHMT, SAHH* and *MTHFD2* and group E of *SHMT1* and *SHMT2*.

**Figure 6:**
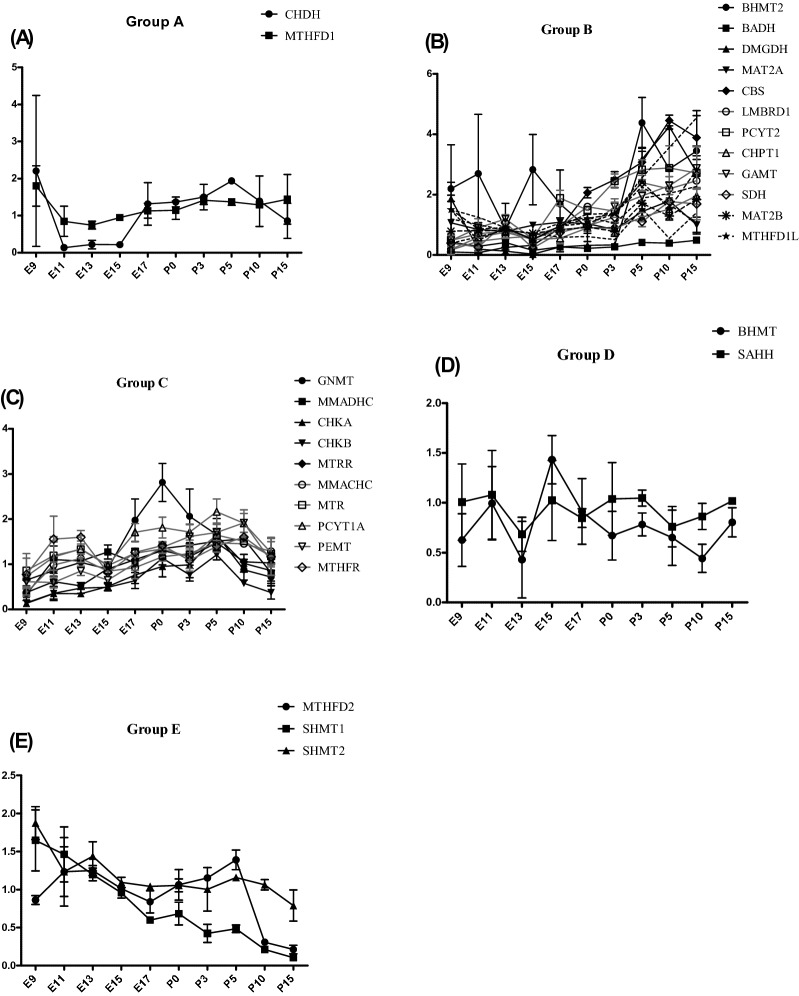
Variation of the expression of 1C metabolism genes in mouse brain ontogenesis. (**A**) Group A genes. (**B**) Group B genes. (**C**) Group C genes. (**D**) Group D genes. (**E**) Group E genes.

Group A gene expression is low until E15 and show a slight increase at E17 (Fig. 6A).

Group B genes have a low expression before birth until E15 and increase slowly from E17 until P10 where they reach a plateau of maximal expression levels (Fig. 6B).

Group C genes have an expression showing a bimodal expression pattern with a first increase between E9 and E11, then a decrease between E13 and E15, and a second increase between E15 and P0 then a decrease until P15 (Fig. 6C).

Group D genes show a relatively stable low expression during ontogenesis (Fig. 6D).

Group E genes present a gene expression that decrease slowly between E9 and P15 (Fig. 6E).

**Figure 7:**
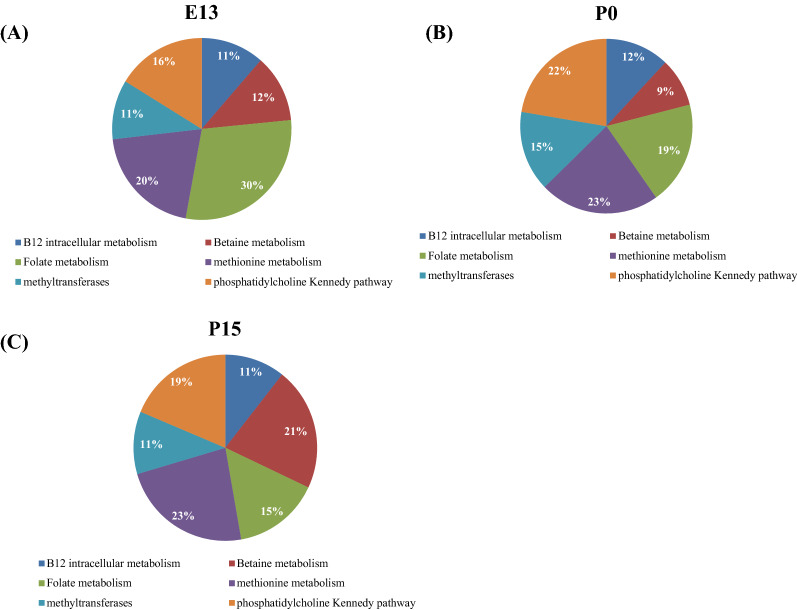
Percentages of brain mRNA expression of genes according to their metabolic pathway at a prenatal stage at embryonic day 13 (**A**), at birth (**B**) and at a postnatal stage, 15 days after birth (**C**).

Figure 7 shows the composition of 1C metabolism genes represented as percentage of the total 1C genes mRNA in a prenatal stage at embryonic day E13 (Fig. 7A), at birth (Fig. 7B) and in a postnatal stage at 15 days of life in brain (Fig. 7C). At E13, the most abundant mRNA belongs to folate metabolism. Between E13 and P0, methionine and phosphatidylcholine metabolisms became the predominant pathways in term of gene expression. Between P0 and P15, mRNA from genes involved in betaine pathway became the most abundant.

## Discussion

The data presented here show the first comprehensive kinetic of the expression of a set of the main genes involved in 1C metabolism in liver and brain throughout pre and postnatal development in OF1 mice.

It is a targeted transcriptomic analysis that includes the gene of the most important metabolic pathways involved in 1-C metabolism. Some other pathways could have been added such as polyamine synthesis however this pathway is considered to consume a minor part of SAM estimated to 5%^18^. The limitation of our study restricted to the transcriptomic level is that mRNA levels may not exactly be related to levels of proteins or enzymes activity. It would be interesting to correlate these data with proteomics and metabolomics data however these methods require a lot of biological material to be robust.

In the literature, the expression of only few genes involved in 1-C metabolism has been studied on limited period during ontegenesis. Li et al*.* have published a global analysis of the liver transcriptome during ontogeny^19^. If we analyze the data regarding the expression of genes involved in 1C metabolism, they get similar results excepted for *GNMT*, *BHMT* and *BHMT2* that show dramatic increases during embryogenesis. However, accurate comparison of the expression of individual genes between studies is difficult because the published data does not exactly match the developmental stages chosen for our study and because Li et al. did not use the same method since they measured the absolute amount of mRNA by high-density oligonucleotide microarrays. Moreover, they use a different mouse strain (C57/B6 mice), and we have observed significant differences of basal expression of genes involved in 1C metabolism depending on the genetic background (Supplemental data 1).

Liver ontogeny initiates at E9 when epithelial cells of the foregut endoderm interact with the cardiogenic mesoderm and commit to becoming the liver primordium. Hematopoietic progenitor cells appear in the liver from day 11 by migration from the yolk sac and regions of the intraembryonic mesoderm^20^. At E10.5–E12.5, liver becomes a major site of fetal hematopoiesis^21^. The maturation of functional hepatocytes and the formation of a biliary network connected to the extrahepatic bile ducts are gradual, beginning at E13 from the bipotential hepatoblasts and continuing until after birth. Hepatoblasts residing next to portal veins become bile epithelial cells while most of the hepatoblasts in the parenchyma differentiate into hepatocytes. As these hepatoblasts gradually become mature hepatocytes the main function of liver switches from hematopoiesis to metabolism. The first bifurcating hierarchy of the cluster we observed in liver distinguishes 2 developmental stages before and after E15. This dichotomy corresponds to the switch of the liver functions from hematopoiesis to metabolism. The liver dendrogram is further divided into 4 sub-stages corresponding to transiting from E13 to E15 and E17 to P0. Previous experiments indicated that E14 is a transition point of mouse liver development around which hepatocytes and bile-duct epithelial cells occurs^21^. The division between E17 and P0 is not surprising since birth occurs between these 2 time points leading to major metabolic adaptation especially for energetic metabolism and macromolecule synthesis^22^. Expression profile between P3 and P15 exhibited the smallest variations suggesting the 1C metabolism in the liver has become near to maturation.

As in other tissues, liver can produce methionine from homocysteine through the ubiquitous methionine synthase (*MTR*), using 5-methyltetrahydrofolate as methyldonor and methylcobalamine as cofactor or through the tissue specific (liver, kidney) BHMT enzyme using betaine as a methyldonor (Fig. 1). While *MTR* and *MTRR* expression remains stable during liver ontogenesis, the expression of *BHMT* and of all genes involved in betaine metabolism (*CHDH*, *BADH*, *DMGDH*, *SDH*) increases regularly during liver ontogenesis suggesting a progressive increased capacity of homocysteine remethylation by this pathway. This is supported by the genes' distribution related to their metabolic pathway, between E13 and P0, the expression of the genes involved in the betaine pathway becomes more preeminent at the expense of the folate metabolism genes (Fig. 4). Together, these data reinforce the idea that the role of betaine in homocysteine remethylation after embryogenesis has been underestimated for a long time.

Methionine is crucial for *S*-adenosylmethionine (SAM) synthesis, since SAM is the methyl donor for almost all methylation reactions (Fig. 1). *MAT1A*, *MAT2A* and *MAT2B* genes encode the different isoforms of methionine adenosyltransferase (MAT) that catalyze the synthesis of SAM. During liver ontogenesis we observed a decreased expression of *MAT2A* and *MAT2B* while expression of *MAT1A* increased from E15. These results are in accordance with the data previously reported in mice and rats indicating that MATII isoform, encoded by *MAT2A* and *MAT2B,* is the major isoform in liver during embryonic life and is progressively replaced by the MATI/III isoforms encoded by *MAT1A*^23,24^.

Liver is also the major tissue for methionine and SAM consumption^25^. We hypothesize that there is an increased consumption of labile methyl groups in the liver from E13 since we observed a continuous increased expression of the three SAM-dependent methyltransferases (e.g. *GNMT*, *PEMT*, *GAMT*) which consume the most of methyl groups.

The methylation reactions using SAM produce *S*-adenosylhomocysteine (SAH) which should be rapidly hydrolyzed into homocysteine as it acts as a potent inhibitor of most of the SAM-dependent methyltransferases. We observed an increased expression of *SAHH* from E17 following the methyltransferases expression increase.

Homocysteine is considered toxic for cells and can only be catabolized through the transsulfuration pathway. *CBS* expression dramatically increase from E15 suggesting an overall increase of catabolism of homocysteine (Fig. 1).

Concerning the hepatic phosphophatidylcholine synthesis, we observed an increased expression of *PEMT*, but expression of *PCYT1A* that codes for the rate limiting enzyme of the Kennedy pathway^26^ remains stable. During embryogenesis, there is an increased demand of phosphatidylcholine for membranes synthesis that might be fulfilled through the PEMT pathway rather than the de novo synthesis.

In cytosol, beside homocysteine remethylation into methionine using 5-methyltetrahydrofolate, other tetrahydrofolates vitamers also serve as 1C donors: 10-formyltetrahydrofolate for purine synthesis and 5,10-methylenetetrahydrofolate for the synthesis of thymidylate. Thymidilate synthesis also occurs in the nucleus for DNA replication and in the mitochondria^27^. Interconversion of tetrahydrofolate vitamers involved different isoenzymes in cytosol and mitochondria encoded by MTHFD1, SHMT1 in the cytosol and nucleus, and by MTHFD2, MTHFD2L, MTHFD1L and SHMT2 in the mitochondria^27,28^. *SHMT2* expression (mitochondrial isoform) increases during embryogenesis while *MTHFD2* and *MTHFD1L* remain stable suggesting that THF is preferentially used for mitochondrial thymidylate synthesis via the production of 5,10-methyleneTHF as it has been recently highlighted in the literature^29^.

1C metabolism is involved in key pathways in brain such as creatine, phospholipids and neurotransmitters synthesis. However, expression of 1C metabolism genes was at a lower level and with patterns that are more variable. It is surprising that gene expressions vary slightly in the brain since the majority of inborn errors of 1C metabolism lead to neurological abnormalities. This suggests that brain 1C metabolism may strongly dependent on other tissues such as the liver. Mouse models and patients suffering from MTHFR deficiency support this hypothesis since the treatment with betaine improve neurological symptoms despite *BHMT* is not expressed in brain^30,31^. After birth, we observed a mild increased expression of the genes involved in the betaine metabolism that become one of the major pathways at E15 (Fig. 7). It has already been suggested that betaine may play additional important roles in brain than homocysteine remethylation like osmotic regulation^32^ as it has been described in the kidney medulla^33^.

In summary, the present study describes mRNA expression patterns of major genes involved in 1C metabolism in liver and brain and provides clues about the methylation demand and methylation pathways in these two tissues during development.

## Supplementary Information

Supplementary Information 1.

Supplementary Information 2.
