# Supplementary material for: Liver and brain differential expression of one-carbon metabolism genes during ontogenesis
Source: Sci Rep. 2021 Oct 26;11:21132. doi: 10.1038/s41598-021-00311-9 (PMC8548596; doi:10.1038/s41598-021-00311-9)
Supplement: Supplementary file 1 — Supplementary Information 1. [file 41598_2021_311_MOESM1_ESM.docx]

**Supplemental data 1 :** **Hepatic level of expression of genes involved in 1C metabolism (alphabetic order) in 6 different mouse strains: 129S2/SVPASCRL, BALB/cByJ, C57BL/6NCRL, DBA/2J, FVB/NCRL and C3H/HeNCrl.**


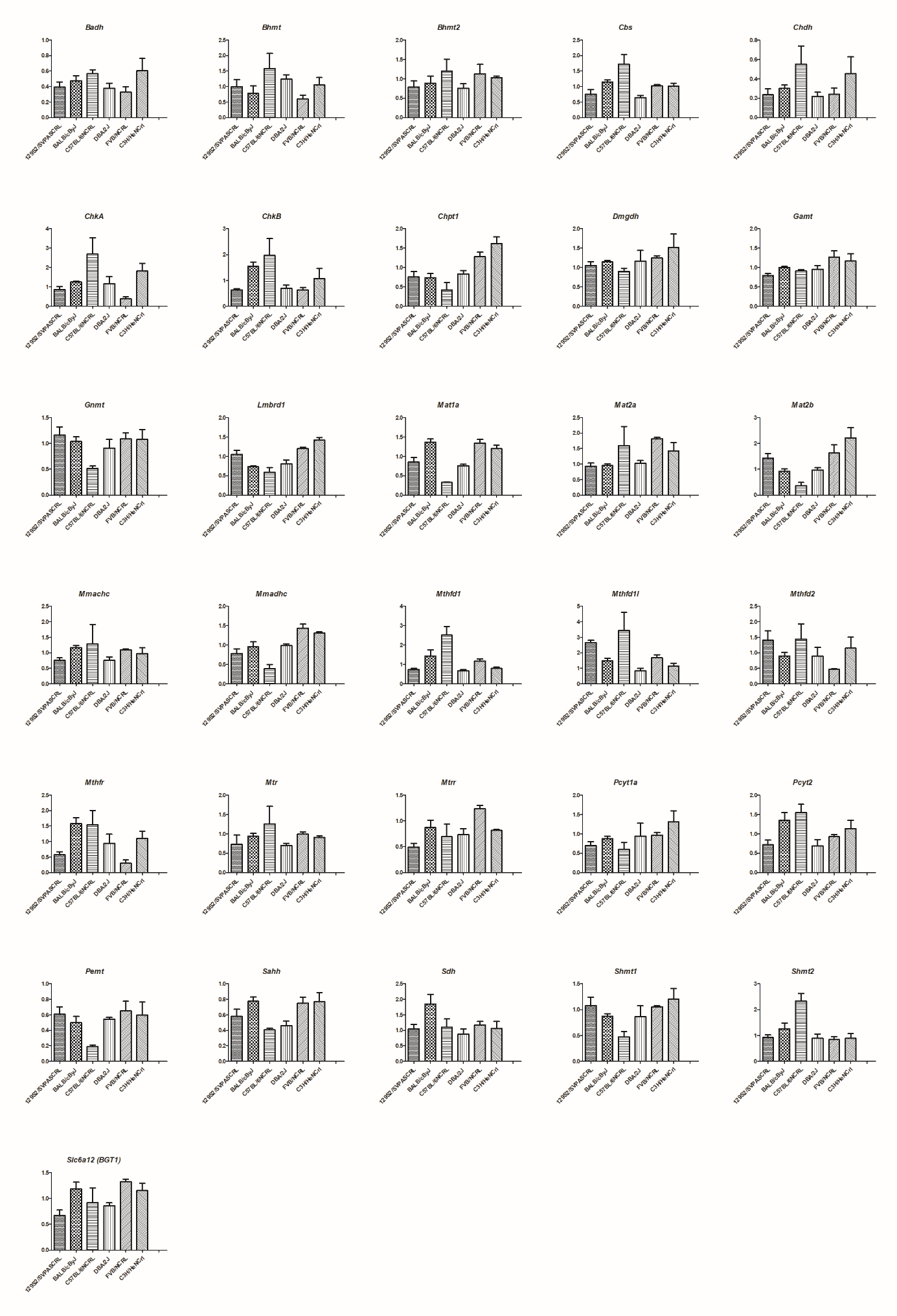


Three female adults of six different mouse strains were used, namely BALB/cByJ, C3H/HeNCrl, DBA/2J, FVB/NCRL, 129S2/SVPASCRL, and C57BL/6NCRL. The animals were fed with standard rodent chow *ad libitum* and sacrificed at six weeks of age. Hepatic level of expression of the 31 genes form 1C metabolism between the 6 mouse strains were expressed in ΔΔCt using GAPDH as reference. The majority of the tested genes showed differences (Kruskall-Wallis test) among the 6 mouse strains that were found significant for *MatA* (p = 0.0187), *Mat2b* (p = 0.0131), *Sahh* (p = 0.0427), *Cbs* (p = 0.0111), *Mmadhc* (p = 0.0129), *Lmbrd1* (p = 0.0106), *ChkaA* (p = 0.0255), *Chpt1* (p = 0.0254), *Mthfr* (p = 0.0411), *Mthfd1* (p = 0.0162), *Mthfd1l* (p= 0.0167).
