# Supplementary material for: Liver and brain differential expression of one-carbon metabolism genes during ontogenesis
Source: Sci Rep. 2021 Oct 26;11:21132. doi: 10.1038/s41598-021-00311-9 (PMC8548596; doi:10.1038/s41598-021-00311-9)
Supplement: Supplementary file 2 — Supplementary Information 2. [file 41598_2021_311_MOESM2_ESM.docx]

| Gene | Forward primer | Reverse primer |
| --- | --- | --- |
| *BADH* | GCTGGCCAGAGGTGTACCA | CCTCATCATGGATGCTTTCATG |
| *BHMT* | CACTCCTGACTGCAACAAGCA | CTGGGTTCCAGTCCAAATGG |
| *BHMT2* | CTGCCAGTGAGGACAATATGGA | GAGGTCACAGGCAGCAGCAT |
| *CBS* | GGGATCGGCTACGACTTCATC | CGTTGCTCTTGAACCACTTGTC |
| *CHDH* | CCTGTGATCCAGCCCAACTAC | GCTTCACACACAGACGGAAATC |
| *CHPT1* | AGTCACTGGATGCTATTGATG | GGATAAGTTCCTAAGCGAGCG |
| *CKA* | GCTCCTGCGGCTGTATGG | GCTTGTTCGGATCCCTCTTTATTA |
| *CKB* | CCAAGTCGGCCATTGAAAAC | TGGCAATGGCTGCTGACA |
| *DDH* | GCCTGGAGAAAGCCTTCAGA | CCAGTCCAGCTTCCAAAGGA |
| *GAMT* | GGAGGAGACCTGGCACACA | GGCGAAAGGCGTGGTTCT |
| *GNMT* | AGCCCGCCTTCGACAAGT | GGCACATCTTTGTCCAGAGTCAT |
| *LMBRD1* | TGTGATGCAGATGCTCCTGAA | CACTGAAGAACCAGAACTTGTGAAG |
| *MAT1* | ATCAAGCACATCGGCTACGAT | GCACGTTGCAAGTCTTGAAGTC |
| *MAT2A* | GTGGGCCTCAGGGTGATG | CCGCCATAAGTGTCCACAATG |
| *MAT2B* | CCCCAGCAGTCACTTAAGACCTA | GAGCATTTCTCGGACGTTGTG |
| *MMACHC* | CCTTTTGGCTTCGAGGTTTACC | GCTGGAGGCAAGAGTTCATTG |
| *MMADHC* | CTGCTGCACCTCCAGATATATGC | TCCAAAGGGTCCCATAGTTTCA |
| *MTHFD1* | AAGTCACTCAGTTGAAGGAGC | GCCTTCAGCTTCACATTTATA |
| *MTHFD1L* | GCAGTGAAGCCGAGATTATA | AGGCATTGAGGACTTTGTTG |
| *MTHFD2* | GCAAGTCACTCCTATGTCCTC | GAGGCCATCTACATTATCATCA |
| *MTHFD2L* | AACAGAAGACCTCACCTCAGTA | CCTTAGGTTTTAGAATGAGCTC |
| *MTHFR* | GGAATTCTTCCCTCCTCGAACT | CCATCCGGTCAAACCTTGAG |
| *MTR* | GTTTTCCTTTACCATGCAATCAAGT | AGGGAGGTTTCCAGCATTCAC |
| *MTRR* | GGCATTTCTATGACACTGGACATG | CCACGGCTCAACCACAAGT |
| *PCYT1A* | CAGCTCCTTTTTCTGATGAA | ATCGGCATAAACTCTCACAG |
| *PCYT1B* | CAGAAACCATGGAGGAAATAG | GTTGGTTTCATCAGCAAATG |
| *PCYT2* | CTAGAGACCCTGGACAAATACA | CTTTACTTCCTCATAGGTGT |
| *PEMT* | CCTTCAATCCGCTCTACTGGAA | CAGCTTGCGGGTCTTGTGT |
| *SAHH* | CTGCCATGGAGGGCTATGAG | CTGTGGTGGTGACAAAGATGTTG |
| *SDH* | CATCGCCTACGGTTACATCCA | GCATAGTCCCCGCTCTTCAC |
| *SHMT1* | GGAGAACGCACGCCTCTTC | TCCAGGTTTCGGGAGTAGCA |
| *SHMT2* | CTGCCAAGCTCCAGGATTTC | TGGCCAGACGCTGACTTGT |

**Table 1: Primers used for quantitative PCR**
